# Chromosome identification and reconstruction of evolutionary rearrangements in *Brachypodium distachyon*, *B. stacei* and *B. hybridum*

**DOI:** 10.1093/aob/mcy086

**Published:** 2018-06-08

**Authors:** Joanna Lusinska, Joanna Majka, Alexander Betekhtin, Karolina Susek, Elzbieta Wolny, Robert Hasterok

**Affiliations:** Department of Plant Anatomy and Cytology, Faculty of Biology and Environmental Protection, University of Silesia in Katowice, Katowice, Poland

**Keywords:** *Brachypodium*, *Brachypodium distachyon*, *Brachypodium hybridum*, *Brachypodium stacei*, chromosome rearrangements, comparative chromosome mapping, dysploidy, karyotype structure and evolution, mcFISH, model grass, nested chromosome fusions, Robertsonian rearrangements

## Abstract

**Background and Aims:**

The *Brachypodium* genus represents a useful model system to study grass genome organization. Palaeogenomic analyses (e.g. Murat F, Armero A, Pont C, Klopp C, Salse J. 2017. Reconstructing the genome of the most recent common ancestor of flowering plants. *Nature Genetics***49**: 490–496) have identified polyploidization and dysploidy as the prime mechanisms driving the diversity of plant karyotypes and nested chromosome fusions (NCFs) crucial for shaping grass chromosomes. This study compares the karyotype structure and evolution in *B. distachyon* (genome Bd), *B. stacei* (genome Bs) and in their putative allotetraploid *B. hybridum* (genomes BdBs).

**Methods:**

*Brachypodium* chromosomes were measured and identified using multicolour fluorescence *in situ* hybridization (mcFISH). For higher resolution, comparative chromosome barcoding was developed using sets of low-repeat, physically mapped *B. distachyon*-derived bacterial artificial chromosome (BAC) clones.

**Key Results:**

All species had rather small chromosomes, and essentially all in the Bs genome were morphometrically indistinguishable. Seven BACs combined with two rDNA-based probes provided unambiguous and reproducible chromosome discrimination. Comparative chromosome barcoding revealed NCFs that contributed to the reduction in the *x* = 12 chromosome number that has been suggested for the intermediate ancestral grass karyotype. Chromosome Bd3 derives from two NCFs of three ancestral chromosomes (Os2, Os8, Os10). Chromosome Bs6 shows an ancient Os8/Os10 NCF, whilst Bs4 represents Os2 only. Chromosome Bd4 originated from a descending dysploidy that involves two NCFs of Os12, Os9 and Os11. The specific distribution of BACs along Bs9 and Bs5, in both *B. stacei* and *B. hybridum*, suggests a Bs genome-specific Robertsonian rearrangement.

**Conclusions:**

mcFISH-based karyotyping identifies all chromosomes in *Brachypodium* annuals. Comparative chromosome barcoding reveals rearrangements responsible for the diverse organization of Bd and Bs genomes and provides new data regarding karyotype evolution since the split of the two diploids. The fact that no chromosome rearrangements were observed in *B. hybridum* compared with the karyotypes of its phylogenetic ancestors suggests prolonged genome stasis after the formation of the allotetraploid.

## INTRODUCTION

*Brachypodium* is a small genus of temperate grasses that belongs to the Brachypodieae tribe within the Pooideae subfamily. It consists of ~20 primarily perennial species that are distributed worldwide, which are quite diverse in terms of their basic chromosome number, nuclear genome size and ploidy level, making their exact phylogenetic relations complex and unresolved (Robertson, 1981; Khan and Stace, 1999; Wolny and Hasterok, 2009; Wolny *et al.*, 2011; Betekhtin *et al.*, 2014). Early chloroplast DNA and internal transcribed spacer (ITS) repeat-based phylogenetic analyses revealed that the *Brachypodium* species emerged soon after the divergence of the Pooideae from Oryzeae (Catalán *et al.*, 1997). The close relationship of *B. distachyon* with economically important temperate cereals and forage grasses combined with many other favourable attributes, such as its very small nuclear genome, simple growth requirements, small stature and rapid annual life cycle, prompted Draper *et al.* (2001) to propose it as a model organism. Although this species was initially used to facilitate functional genomics analyses in grasses, other useful features rapidly promoted its use in diverse research programmes and ensured a continuous growth of various experimental tools and resources, such as large germplasm collections, sequenced (IBI, 2010) and resequenced (Gordon *et al.*, 2014) genomes, cDNA libraries (Gordon *et al.*, 2014), high-coverage genomic DNA libraries of ordered bacterial artificial chromosome (BAC) clones (Febrer *et al.*, 2010) and efficient mutagenesis and transformation protocols (Vogel and Hill, 2008).

Based on simple cytogenetic analyses, Robertson (1981) postulated the presence of three *B. distachyon* cytotypes that had diploid chromosome numbers of 2*n* = 10, 20 and 30 and that constituted an autopolyploid series with a base chromosome number of *x* = 5. Later, the use of genomic DNA- and BAC-based probes in fluorescence *in situ* hybridization (FISH) analyses gave clear indications that these cytotypes may represent distinct species (Hasterok *et al*., 2004, 2006*a*, *b*). Combining phenotypic, cytogenetic and molecular studies, Catalán *et al.* (2012) proposed that the cytotypes be classified as two separate diploid species, i.e. *B. distachyon* (2*n* = 10, genome Bd) and *B. stacei* (2*n* = 20, genome Bs), and their natural allotetraploid *B. hybridum* (2*n* = 30, genomes BdBs), which are the only annuals in the genus. The distinction of the new species was corroborated by DNA barcoding (Lopez-Alvarez *et al.*, 2012), and more recently by the successful resynthesis of stable and fertile *B. hybridum* from an interspecific cross between *B. distachyon* and *B. stacei*, which closely resembles its natural counterpart (Dinh Thi *et al.*, 2016). *Brachypodium stacei* and *B. hybridum* have now attracted increasing research interest and their genome sequencing projects are nearing completion (https://jgi.doe.gov/our-science/science-programs/plant-genomics/brachypodium).

Many cytological features and existing resources make *B. distachyon* and other species in the genus exceptionally useful model systems in various areas of plant molecular cytogenetics (e.g. Wolny *et al.*, 2014; Idziak *et al.*, 2015; Borowska-Zuchowska and Hasterok, 2017; Kus *et al.*, 2017). The availability of the whole genome sequence of *B. distachyon* (IBI, 2010) and its combination (Febrer *et al.*, 2010) with efficient BAC–FISH-based methodology (Hasterok *et al*., 2006*a*, *b*; Jenkins and Hasterok, 2007) enabled the first true chromosome painting in monocotyledonous plants (Idziak *et al.*, 2011; Betekhtin *et al.*, 2014; Robaszkiewicz *et al.*, 2016). This approach is especially effective in visualizing specific chromosomes and large segments, particularly in long pachytene chromosomes, which has helped to elucidate the mechanisms of karyotype evolution in *Arabidopsis thaliana* and some other Brassicaceae (Lysak *et al*., 2006, 2010) and, more recently, in *Cucumis* (Lou *et al.*, 2014; Han *et al.*, 2015). Chromosome barcoding is a complementary approach that exploits either single low-repeat BACs or their small pools. By precisely targeting specific chromosome regions, it allows even relatively small rearrangements, such as duplications, deletions and inversions, to be detected (Szinay *et al.*, 2012; Hoang and Schubert, 2017; Tran *et al.*, 2017). In *Brachypodium*, the *B. distachyon*-derived BACs hybridize across the genus and facilitate effective mapping on mitotic chromosome preparations. Until now, this approach has been used for individual chromosome discrimination and karyotyping in the diploid *B. pinnatum* (2*n* = 18) (Wolny *et al.*, 2013) and on a small scale to analyse chromosome structure and evolution in some *Brachypodium* perennials (Wolny *et al.*, 2011; Idziak *et al.*, 2014).

Synteny-based reconstructions of ancient angiosperm genomes imply that the genomes of extant species originated from ancestral genomes that had the lowest number of historical polyploidization events (Abrouk *et al.*, 2010). Although this provided insight into the putative numbers of protochromosomes in the monocot progenitors and permitted the karyotypes of some present-day grasses, including this of *B. distachyon*, to be connected with their hypothetical ancestral karyotypes, these studies did not involve other *Brachypodium* representatives (IBI, 2010; Salse, 2016*a*, *b*). Moreover, cytomolecular data about karyotype organization in *B. stacei* and *B. hybridum* are limited, since they have considerably more chromosomes than *B. distachyon*, the vast majority of which were until recently unidentifiable.

Here we present the holistic characteristics of the *B. distachyon*, *B. stacei* and *B. hybridum* karyotypes using a general morphometric analysis of chromosomes together with their unambiguous and reproducible identification using multicolour FISH (mcFISH) with chromosome-specific probes. In order to gain more detailed insight into the karyotype structure and evolution of *B. stacei* and *B. hybridum*, we also used comparative chromosome barcoding (CCB) with a series of single low-repeat BAC clones that were derived from chromosomes Bd3 and Bd4 of *B. distachyon*.

## MATERIALS AND METHODS

### Plant material

Seven genotypes of the three *Brachypodium* species were used in this study. Their names, basic cytogenetic characteristics and other essential information are provided in Table 1.

**Table 1.** General characteristics of the *Brachypodium* species that were used in this study

Species	Accession number	2*n*	*x*	Genome designation	Genome size (pg/2C DNA)	Origin	Source
*B. distachyon*	Bd21	10	5	Bd	0.631	Iraq	a
*B. stacei*	ABR114	20	10	Bs	0.564	Spain, Formentera	b
	ABR200	20	10	Bs	n/a	n/a	b
	Bsta5	20	10	Bs	n/a	Spain, Alicante	c
*B. hybridum*	ABR113	30	5 + 10	BdBs	1.265	Portugal, Lisbon	b
	ABR100	30	5 + 10	BdBs	n/a	Iran, Khalaf Abad	b
	ABR117	30	5 + 10	BdBs	n/a	Afghanistan	b

Source: a, US Department of Agriculture, National Plant Germplasm System, USA; b, Institute of Biological, Environmental and Rural Sciences, Aberystwyth University, Aberystwyth, UK; c, High Polytechnic School of Huesca, University of Zaragoza, Huesca, Spain.

Genome size data from Wolny and Hasterok (2009) and Catalán *et al.* (2012). n/a, not available.

### Chromosome preparation

Multisubstrate chromosome preparations were made according to the methodology of Hasterok *et al.* (2006*a*) with some modifications. Briefly, Petri dishes with 3- to 4-d-old seedlings were placed in a box with ice for 24 h. Whole seedlings were fixed in 3:1 (v/v) 100 % methanol/glacial acetic acid at 4 °C for a minimum of 3 h or overnight and stored at −20 °C until used. *B. distachyon* and *B. hybridum* roots were digested in an enzyme mixture consisting of 8 % (v/v) pectinase (Sigma), 1 % (w/v) cellulase (Sigma) and 1 % (w/v) cellulase Onozuka R-10 (Serva) in a 0.01 m citric acid–sodium citrate buffer (pH 4.8) for 2 h at 37 °C. For the roots of *B. stacei*, the concentrations of these enzymes were 6, 0.5 and 0.5 %, respectively, with the digestion time extended to 2 h 40 min at 37 °C.

### Clone selection, probe labelling and FISH

All of the BAC clones originated from the BD_ABa and BD_CBa genomic DNA libraries and were derived from the FingerPrinted Contigs that had previously been assigned to chromosomes Bd1–Bd5 of *B. distachyon* Bd21 (Febrer *et al.*, 2010). The clones were selected on the basis of their even distribution along the entire length of a given chromosome. With the exception of the centromeric clone BD_CBa0033J12, in order to minimize the risk of unwanted cross-hybridization, only low-repeat (i.e. containing <23 % of repetitive sequences) BACs were selected (Table 2) using RepeatMasker (http://www.repeatmasker.org;Febrer *et al.*, 2010). With the aim of determining the inter-individual consistency, each clone was mapped to chromosome preparations of at least seven individuals of each species and three different accessions of *B. stacei* and *B. hybridum* (Table 1).

**Table 2.** Specification of the BAC clones that were used in the FISH analyses

Chromosome marker	BAC name	BAC clone identifier*	Position in the genome (bp)
Centromere visualization	CEN	BD_CBa0033J12	–
	Bd1S	BD_CBa0030L10^§^	Bd1: 8680898: 8845282
	Bd2S/1	BD_ABa0005E09^§†^	Bd2: 10380990: 10507985
Chromosome identification	Bd2S/2	BD_CBa0023P23^†^	Bd2: 13336480: 13486307
	Bd3S	BD_CBa0014A01^§†^	Bd3: 20363699: 20508591
	Bd3L	BD_ABa0019B17^†^	Bd3: 50354409: 50508627
	Bd4S	BD_CBa0022F16^§^	Bd4: 1375208: 1513070
	Bd3S/1	BD_CBa0028O16^§^	Bd3: 856255: 1007650
	Bd3S/2	BD_ABa0015A18^§^	Bd3: 4001904: 4157452
	Bd3S/3	BD_ABa0018B12^§^	Bd3: 6003300: 6153924
	Bd3S/4	BD_ABa0030J22^§^	Bd3: 8504730: 8651070
	Bd3S/5	BD_CBa0016A22^§^	Bd3: 11505050: 11712720
	Bd3S/6	BD_ABa0022G01^§^	Bd3: 16038657: 16055486
	Bd3S/7	BD_ABa0033D16^§^	Bd3: 22106200: 22299788
	Bd3L/8	BD_CBa0011M04^§^	Bd3: 36854229: 37002472
	Bd3L/9	BD_ABa0038N13^§^	Bd3: 44001051: 44142538
	Bd3L/10	BD_ABa0026M18^§^	Bd3: 49347850: 49503810
	Bd3L/11	BD_ABa0037F15^§^	Bd3: 50854746: 51004145
Chromosome structure and evolution	Bd3L/12	BD_ABa0037C10^§^	Bd3: 52501229: 52687354
	Bd3L/13	BD_ABa0008G22^§^	Bd3: 55503533: 55665230
	Bd3L/14	BD_ABa0020N10^§^	Bd3: 57504387: 57653389
	Bd4S/1	BD_CBa0030B12^§^	Bd4: 2007584: 2157984
	Bd4S/2	BD_CBa0040J03^§^	Bd4: 7830905: 8001843
	Bd4S/3	BD_CBa0021B09^§^	Bd4: 9502901: 9667864
	Bd4S/4	BD_ABa0043D11^§^	Bd4: 11006774: 11150531
	Bd4S/5	BD_ABa0010I18^§^	Bd4: 14002249: 14164264
	Bd4L/6	BD_ABa0006J17^§^	Bd4: 29358544: 29516826
	Bd4L/7	BD_ABa0020D08^§^	Bd4: 32504625: 32642850
	Bd4L/8	BD_CBa0035E05^§^	Bd4: 39350118: 39526113
	Bd4L/9	BD_CBa0038H23^§^	Bd4: 42789149: 43003220
	Bd4L/10	BD_ABa0041I03^§^	Bd4: 48350055: 48507632

*More details can be found in the NCBI database at the URLs http://www.ncbi.nlm.nih.gov/clone/library/genomic/424 (BD_ABa library) and http://www.ncbi.nlm.nih.gov/clone/library/genomic/426 (BD_CBa library).

^§^Clones used in pools for comparative chromosome painting by Idziak *et al.* (2011) and Betekhtin *et al.* (2014).

^†^Single clones used for CCB-based mapping by Idziak *et al.* (2014).

The BAC DNA was isolated using the standard alkaline lysis method, and then labelled by nick-translation with tetramethylrhodamine-5-dUTP (Roche), digoxigenin-11-dUTP (Roche) or biotin (Roche). The nick-translated 25S ribosomal DNA (rDNA) and 5S rDNA probes were prepared using the clone that contained a 2.3-kb ClaI fragment of the 25S rRNA gene of *A. thaliana* (Unfried and Gruendler, 1990) and the clone pTa794, which contained the 5S rRNA gene from wheat (Gerlach and Dyer, 1980), respectively.

The FISH procedure and kinetic conditions followed Jenkins and Hasterok (2007) with minor modifications. For CCB, the hybridization mixture consisted of 30 % deionized formamide, 40 % dextran sulphate, 2 × SSC, 0.5 % SDS and 50–100 ng mL^–1^ of each probe DNA. Post-hybridization washes were equivalent to ~60 % stringency. The hybridization signals were detected using green fluorescein isothiocyanate (FITC)-conjugated anti-digoxigenin antibodies (Roche), far red- (false-coloured to yellow) Alexa Fluor 647-conjugated anti-biotin antibodies (Jackson ImmunoResearch), or, in the case of red tetramethylrhodamine-5-dUTP, directly visualized. Air-dried preparations were mounted and counterstained in Vectashield (Vector Laboratories) containing 2.5 µg mL^−1^ of DAPI (4′,6-diamidino-2-phenylindole; Serva). For karyotype analysis, slide reprobing was performed as previously described (Schwarzacher and Heslop-Harrison, 2000).

Photomicrographs were acquired using either an AxioImager.Z.2 (Zeiss) or Provis AX (Olympus) wide-field epifluorescence microscope equipped with high-sensitivity monochromatic cameras (AxioCam Mrm [Zeiss] and Retiga 2000R [QImaging], respectively) and the respective narrow-band filter sets. All of the images were then digitally coloured using Wasabi (Hamamatsu Photonics), uniformly processed (if required) to improve contrast and brightness, and then superimposed using Photoshop CS3 (Adobe) and ImageJ (NIH).

### Chromosome measurements

**Fig. 1.**
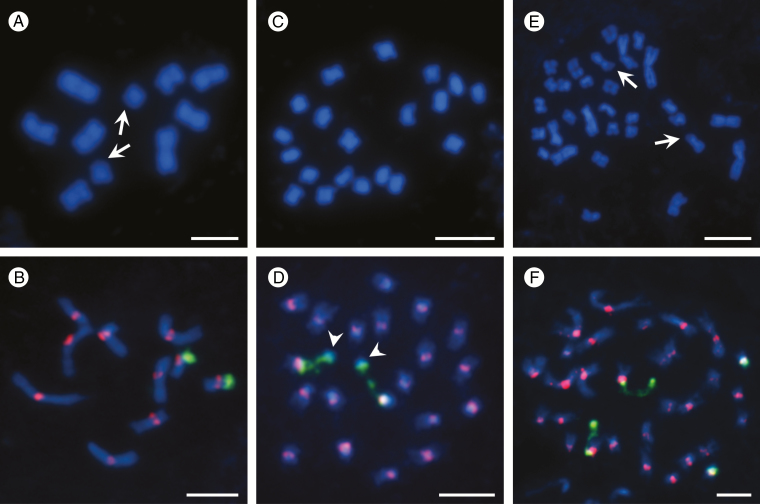
Somatic metaphase (A, C, E) and prometaphase (B, D, F) chromosomes of *B. distachyon* Bd21 (A, B), *B. stacei* ABR114 (C, D) and *B. hybridum* ABR113 (E, F). Chromosomes in (A, C, E) were stained with DAPI (blue) only, arrows in (A, E) show DAPI-negative secondary constriction/satellite regions. Chromosomes in (B, D, F) were subjected to FISH using centromeric (red) and 25S rDNA (green) probes. Arrowheads in (D) show the specific organization of the satellites in *B. stacei*. Scale bars = 5 µm.

**Fig. 2.**
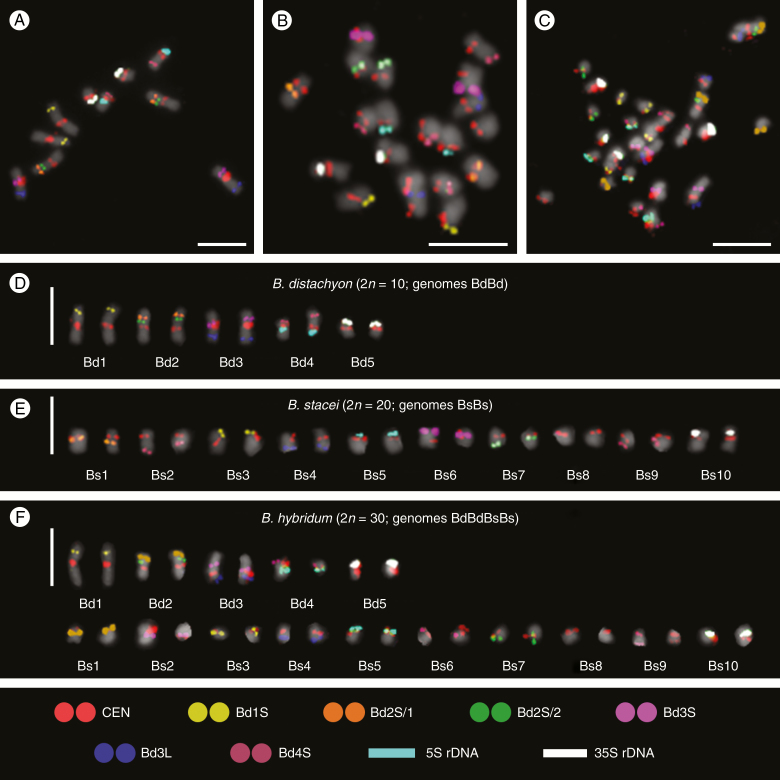
Multicolour FISH identification of *B. distachyon* Bd21 (A, D), *B. stacei* ABR114 (B, E) and *B. hybridum* ABR113 (C, F) somatic metaphase chromosomes using nine probes that were sequentially hybridized to the same preparations. Chromosomes in the karyograms (D, E, F) were extracted from the respective photomicrographs (A, B, C) and ordered according to their total descending length with the 35S rDNA-bearing chromosomes placed at the end, regardless of their length. All of the morphometric chromosome parameters are detailed in Supplementary Data Tables S1–S3. Chromosomes were stained with DAPI (grey). Scale bars = 5 µm.

**Fig. 3.**
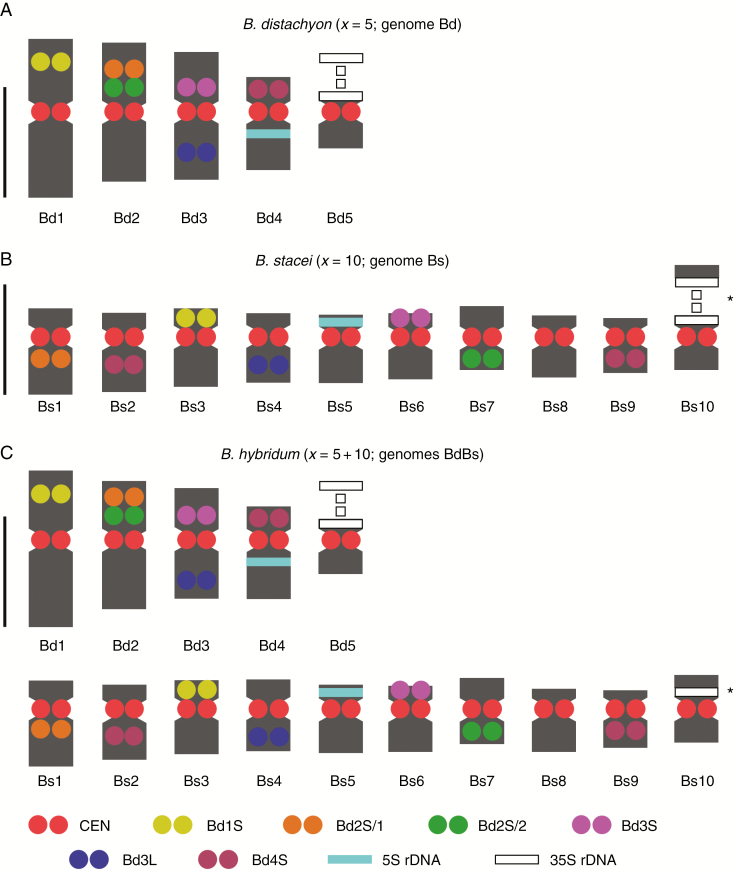
Ideograms of *B. distachyon* Bd21 (A), *B. stacei* ABR114 (B) and *B. hybridum* ABR113 (C) somatic metaphase chromosomes. The ideogram in (C) represents the sum of those in (A) and (B) except for some minor variation regarding Bs10 chromosomes, which is connected with a difference in condensation of their 35S rDNA loci (marked by asterisks). The lengths and shapes of the chromosomes in the diagrams are based on the mean morphometric parameters from Supplementary Data Tables S1–S3. Scale bars = 2.5 µm.

Chromosomes were measured using MicroMeasure 3.3 (Reeves, 2001), which allowed calculation of their total length (S+L), the lengths of the short (S) and long (L) arms, and the arm length ratio (L/S AR). We used FISH with the centromeric clone BD_CBa0033J12 and the 25S rDNA probe to improve visualization of the primary constrictions and 35S rDNA-bearing chromosomes, respectively (Fig. 1). The classification and nomenclature of chromosome types was based on the AR and was adopted from Levan *et al.* (1964) with minor modifications. According to this criterion, all of the chromosomes were identified as either metacentric (m type; L/S AR = 1.01–1.7) or submetacentric (sm type; L/S AR = 1.71–3.0). The calculations that were performed for individual mitotic chromosome spreads and the mean values for the three *Brachypodium* species are presented in Supplementary Data Tables S1–S3. The lengths and shapes of the chromosomes in the ideograms are based on the values that were averaged for the respective pairs of homologous chromosomes (Figs 2 and 3) and are shown Supplementary Data Tables S1–S3.

## RESULTS

### Basic morphometry of chromosomes

The karyotype of *B. distachyon* at mitotic metaphase or prometaphase was the most variable in terms of both chromosome length and morphology and consisted of five chromosome pairs (Bd1–Bd5) with the mean length of the longest chromosome Bd1 equal to 3.6 µm and the mean length of the shortest chromosome Bd5 equal to 1.3 µm. The three longest chromosome pairs (Bd1–Bd3) were metacentric with an average L/S AR that ranged from 1.0 to 1.15, while chromosome pairs Bd4 and Bd5 were submetacentric with L/S AR 1.8 and 2.25, respectively (Supplementary Data Table S1). A characteristic feature of Bd5 was its secondary constriction and a satellite that was located terminally on the short arm. These were sometimes distinguishable as more or less prominent negative chromosome regions after DAPI staining (Fig. 1A; arrows). FISH with the 25S rDNA probe showed that the 35S rDNA locus in this region involved the proximal part of the Bd5 short arm with a secondary constriction and the entire satellite (Fig. 1B).

The 20 chromosomes in the root-tip cells of *B. stacei* (Fig. 1C) were much smaller and more morphologically uniform than those of *B. distachyon*. The mean length of the longest mitotic metaphase chromosome was 1.95 µm, whereas the shortest one was only 1.25 µm. Most of them were submetacentric with an average L/S AR of 1.73–2.44, with only one pair of metacentrics having an L/S AR equal to 1.0 (Supplementary Data Table S2). The only 35S rDNA locus in this genome had a secondary constriction that is often distended. However, unlike its counterpart in *B. distachyon*, it had a visibly more prominent and DAPI-positive satellite, the distal part of which had no rDNA signal (Fig. 1D; arrowheads).

The somatic chromosome complement of *B. hybridum* comprised 30 chromosomes, which differed significantly in their lengths and shapes (Fig. 1E). The mean length of the longest chromosome was 3.55 µm versus only 1.25 µm for the shortest chromosome. Submetacentrics (average L/S AR 1.72–2.25) were predominant in this karyotype over metacentrics (average L/S AR between 1.13 and 1.41), which constituted only one-third of all of the chromosomes (Supplementary Data Table S3). In view of the hybrid nature of this species, all of the large chromosomes originated from the putative *B. distachyon* parent, and the vast majority of the small chromosomes came from the *B. stacei* parent. *Brachypodium hybridum* ABR113 had two pairs of 35S rDNA loci in its somatic chromosome complement, one of which was significantly more decondensed during mitosis than the other (Fig. 1E [arrows], F).

### Multicolour FISH karyotyping

Our aim was to unambiguously identify all of the chromosomes in the complements of the species under study using the smallest possible number of FISH probes. Thus, we selected six low-repeat BACs (Table 2) and two different (25S and 5S) rDNA probes. These were complemented by the centromeric BAC, which provided more reliable determination of chromosome shape and better discrimination of the chromosome arms, particularly in the case of the metacentric chromosomes of the Bd genome and the small chromosomes of the Bs genome (Fig. 2). Since only two or three probes were simultaneously visualized in one FISH experiment using green, red and far red fluorescence, it was necessary to apply to the same preparation four successive FISH rounds using probe combinations Bd2S/1 + Bd2S/2, Bd3S + Bd1S, CEN + 25S rDNA + 5S rDNA and Bd3L + Bd4S, and to use false colours during image processing.

All six low-repeat BACs used for the karyotyping yielded reproducible signals and enabled the discrimination of all chromosomes in the complements of all species in this study (Fig. 2A–C). This allowed us to propose for the first time the nomenclature (Bs1–Bs10) for *B. stacei* and *B. hybridum*, and to arrange the chromosomes of Bs into karyograms (Fig. 2E, F) and ideograms (Fig. 3B, C). All karyograms and ideograms in this study are based not only on the distribution of the mcFISH markers but also on the averaged morphometric parameters (Supplementary Data Tables S1–S3) of chromosomes shown in Fig. 2A–C.

The clone Bd1S (yellow in Figs 2 and 3) marked the distal part of the short arm of chromosome Bd1 in *B. distachyon* (Fig. 2D), but in *B. stacei* was diagnostic for the terminal part of the short arm of chromosome Bs3 (Figs 2E and 3B). There were two clones that hybridized to the short arm of chromosome Bd2 (Figs 2D and 3A), and one of these (Bd2S/1, orange) was more distally located than the other (Bd2S/2, green). In *B. stacei*, Bd2S/1 marked the proximal part of the long arm of chromosome Bs1, while Bd2S/2 was specific to the terminal part of the corresponding arm of Bs7. Two BACs were specific to the Bd3 chromosome; while Bd3S (purple) marked the proximal part of its short arm, Bd3L (blue) occupied the intercalary part of the long arm (Figs 2D and 3A). In the *B. stacei* genome, the first of the clones produced a distinct signal in the terminal part of the short arm of Bs6 and the second clone specifically marked the Bs4 chromosome in the distal part of its long arm (Figs 2E and 3B). Interestingly, clone Bd4S (magenta), which was located rather distally on the short arm of chromosome Bd4 in *B. distachyon* (Figs 2D and 3A), was not completely chromosome-specific in *B. stacei*, where it hybridized with the long arms of chromosomes Bs2 and Bs9 (Figs 2E and 3B). Additionally, the 5S rDNA probe (cyan), which highlighted the only locus for these genes in the *B. distachyon* genome in the proximal region of Bd4 (Figs 2D and 3A), permitted the specific detection of chromosome Bs5 in *B. stacei*, where it hybridized to the subterminal part of its short arm (Figs 2E and 3B). Finally, the Bd5-marking (Figs 2D and 3A) 25S rDNA probe (white) discriminated the short arm of chromosome Bs10 (Figs 2E and 3B).

We also applied the same set of probes to the *B. hybridum* chromosomes (Fig. 2C). This experiment revealed that the chromosome complement of this species is a perfect combination of those of *B. distachyon* and *B. stacei*, not only with respect to chromosome numbers and shapes but also in terms of the distribution of all of the mcFISH landmarks (Figs 2F and 3C).

### Comparative chromosome barcoding

**Fig. 4.**
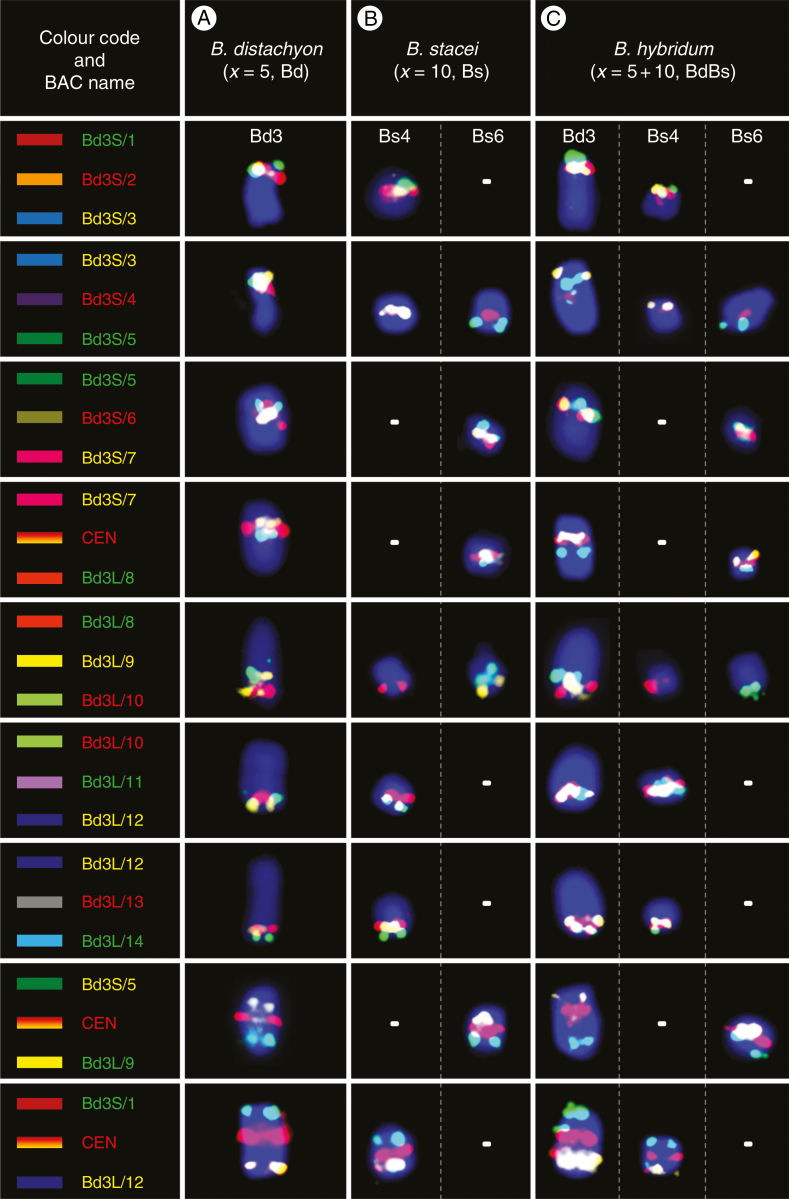
BAC–FISH-based comparative chromosome barcoding with the clones from chromosome Bd3 of *B. distachyon* (A) to chromosomes Bs4 and Bs6 of *B. stacei* (B) and Bd3, Bs4 and Bs6 of *B. hybridum* (C). Only one homologue from a pair is shown. The BAC name text labels in the first column indicate the fluorochrome that was used (green, FITC; red, tetramethylrhodamine; yellow [false colour], Alexa Fluor 647). The chromosomes were stained with DAPI (blue). The coloured bars on the left and BAC names that were assigned to specific clones correspond to those on the cytogenetic maps in Fig. 5.

**Fig. 5.**
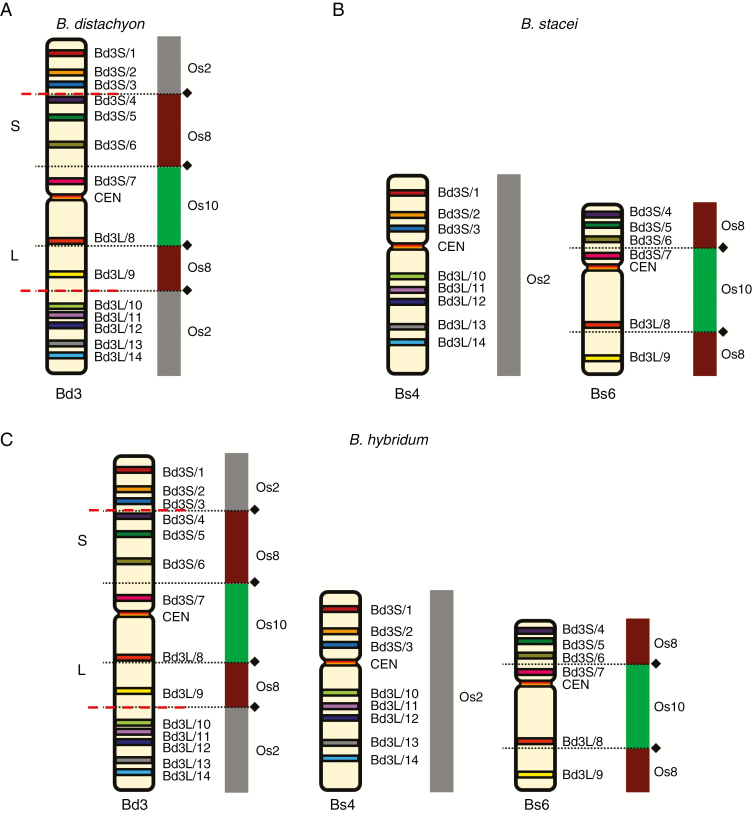
Chromosomal distribution of the BAC clones that were derived from chromosome Bd3 of *B. distachyon* (A) and comparatively mapped to the chromosomes of *B. stacei* (B) and *B. hybridum* (C). The distribution of the clones on the chromosome diagram (A) reflects their position on the physical map of the *B. distachyon* genome (Febrer *et al.*, 2010). The diagrams next to the *Brachypodium* chromosomes relate the BAC clones to the homoeologous regions in different rice chromosome equivalents. Black diamonds and dotted lines indicate the hypothetical fusion points of the intermediate ancestral grass chromosomes in Bd3 (adapted from IBI, 2010). Red dashed lines indicate the chromosomal breakpoints that were found in the Bs-genome chromosomes in *B. stacei* and *B. hybridum*.

**Fig. 6.**
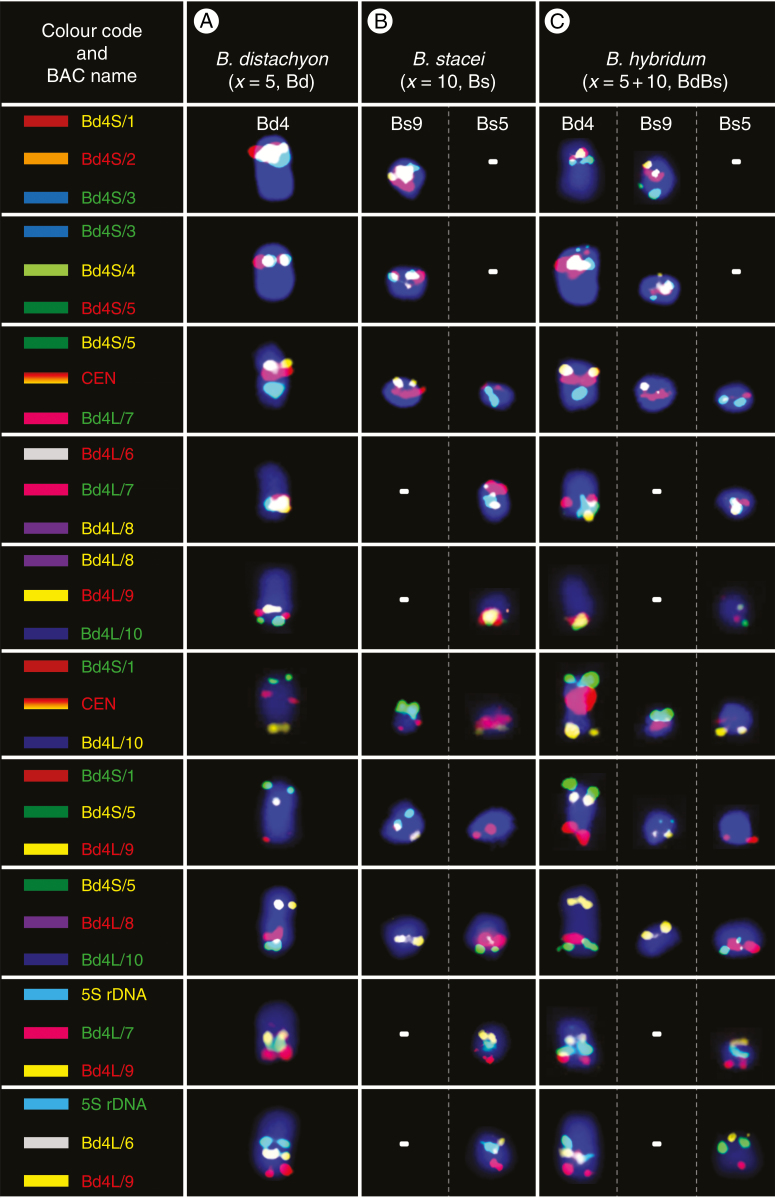
BAC–FISH-based comparative chromosome barcoding with the clones from chromosome Bd4 of *B. distachyon* (A) to chromosomes Bs9 and Bs5 of *B. stacei* (B) and Bd4, Bs9 and Bs5 of *B. hybridum* (C). Only one homologue from a pair is shown. The red, yellow and green BAC name text labels in the first column indicate the fluorochrome used (green, FITC; red, tetramethylrhodamine; yellow [false colour], Alexa Fluor 647). Chromosomes were stained with DAPI (blue). The coloured bars on the left and BAC names that were assigned to specific clones correspond to those on cytogenetic maps in Fig. 7.

**Fig. 7.**
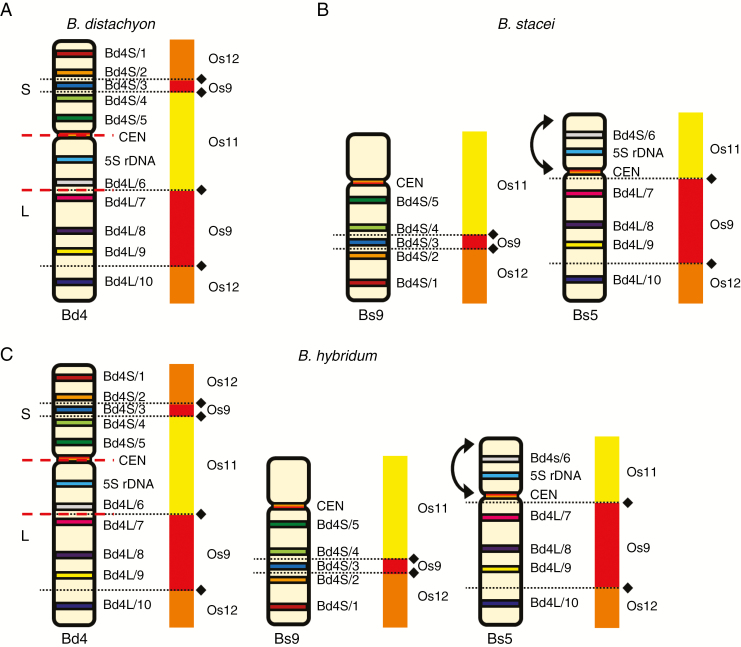
Chromosomal distribution of the BAC clones that were derived from chromosome Bd4 of *B. distachyon* (A) and comparatively mapped to the chromosomes of *B. stacei* (B) and *B. hybridum* (C). The distribution of the clones on the chromosome diagram (A) reflects their position on the physical map of the *B. distachyon* genome (Febrer *et al.*, 2010). The diagrams next to the *Brachypodium* chromosomes relate the BAC clones to homoeologous regions in different rice chromosome equivalents. Black diamonds and dotted lines indicate the hypothetical fusion points of the intermediate ancestral grass chromosomes in Bd4 (adapted from IBI, 2010). Red dashed lines indicate the chromosomal breakpoints that were found in the Bs-genome chromosomes in *B. stacei* and *B. hybridum*. Arrows point to the inversion that was present on chromosomes Bs5 of these species.

For detailed comparative analyses of the structure and evolution of the chromosomes in the three *Brachypodium* annuals, we selected an additional 24 low-repeat BAC clones, 14 of which were derived from chromosome Bd3 and ten from Bd4 and spanned these two chromosomes uniformly along their entire length (Table 2) (Febrer *et al.*, 2010). For most FISH experiments, overlapping triplets of clones that occupied adjacent positions on the physical map of a given chromosome were used together as sets of differentially labelled probes. These were complemented by several additional probe combinations as well as with the centromeric BAC clone, which were useful in confirming some mapping details. Firstly, we verified that the distribution of all of the clones along chromosomes Bd3 and Bd4 (Figs 4A and 6A, respectively) in *B. distachyon* was consistent with their expected position on the physical map (Table 2). Then, we performed cross-species chromosome mapping, which revealed that all of the clones gave single-locus FISH signals on the chromosomes of the Bs genome that were homoeologous to Bd3 (chromosomes Bs4 and Bs6; Figs 4B and 5B) and Bd4 (chromosomes Bs9 and Bs5; Figs 6B and 7B), respectively. In *B. hybridum*, the number and distribution of all of the clones were always the composite of the two other species (Figs 4–7), which is consistent with the putative origin of this allotetraploid. The intensity of the hybridization signals on the Bs-genome chromosomes in both *B. stacei* and *B. hybridum* was often visibly weaker than on the chromosomes belonging to the Bd genome in *B. distachyon* and in *B. hybridum*, but was sufficient to permit analysis. The number of chromosomes carrying the signals of the BACs that occupied adjacent positions on the respective Bd chromosomes (Figs 4A and 6A) was one or two in the genome Bs of *B. stacei* (Figs 4B and 5B) and two or three in the Bd and Bs genomes in *B. hybridum* (Figs 4C and 6C). We did not observe any intraspecific differences in the BAC–FISH signal number and distribution among the three different accessions of *B. stacei* and *B. hybridum* (Supplementary Data Fig. S1).

### Mapping the Bd3-derived clones

Among the clones that had originated from chromosome Bd3 of *B. distachyon*, Bd3S/1 and Bd3L/14 mapped most distally to its short and long arms, respectively (Figs 4A and 5A). In *B. stacei* (Figs 4B and 5B), clones Bd3S/1–3 hybridized to the distal part of the short arm on one chromosome, which by linking our mcFISH-based karyotype with the physical map information was identified as Bs4. Within the next triplet of BACs, starting with clone Bd3S/3 (mapped in yellow to Bs4), the other two, i.e. Bd3S/4 (red) and Bd3S/5 (green), were on another *B. stacei* chromosome, which was identified as Bs6 by karyotyping. It should be noted that, due to its repetitive content, Bd3S/4 also had weak cross-hybridization to the centromeric regions of all Bs chromosomes but its most prominent signal was always detected in the close vicinity of Bd3S/5. Next, two sets of probes (Bd3S/5–7 and Bd3S/7 + CEN + Bd3L/8) were characterized by an undisrupted linear arrangement along the interstitial part of chromosome Bs6. Then, within the next triplet of FISH probes, the first two clones, i.e. Bd3L/8 and 9, still mapped on Bs6 towards the distal part of its long arm. By contrast, Bd3L/10 was localized in the interstitial part of the long arm of Bs4, and was followed by the four remaining clones (Bd3L/11–14) to the end of its long arm in a linear order. Two additional probe combinations that consisted of distantly localized clones – Bd3S/5, Bd3L9 and Bd3S/1, Bd3L/12, which were complemented by the centromeric BAC – confirmed that all of the Bd3-derived clones were restricted in their mapping to only two chromosomes in the *B. stacei* genome, i.e. Bs4 and Bs6. Mapping all of the Bd3-derived clones in *B. hybridum* (Figs 4C and 5C) showed, in terms of FISH signal number, distribution and intensity, that they represented a simple sum of those that were observed in *B. distachyon* (Figs 4A and 5A) and *B. stacei* (Figs 4B and 5B).

### Mapping the Bd4-derived clones

Similarly to Bd3, clone Bd4S/1 mapped most distally to the short arm of chromosome Bd4 of *B. distachyon*, and Bd4L/10 was at the opposite chromosomal end (Figs 6A and 7A). Comparative mapping revealed that clones Bd4S/1–5, derived from the short arm, and clones Bd4L/7–10, from the long arm of Bd4, hybridized consistently to two different chromosomes in *B. stacei* that were identified as Bs9 and Bs5, respectively (Figs 6B and 7B). Interestingly, clone Bd4L/6, which was located on the long arm in the vicinity of the 5S rDNA locus in *B. distachyon*, was still closely connected with this rDNA locus in Bd5, although it gave a hybridization signal in the distal part of the short arm. Additional mapping experiments using five probe combinations (Bd4S/1 + CEN + Bd4L/10, Bd4S/1 + Bd4S/5 + Bd4L/9, Bd4S/5 + Bd4S/8 + Bd4L/10, 5S rDNA + Bd4L/7 + Bd4L/9 and 5S rDNA + Bd4L/6 + Bd4L/9) indicated that the structure of the Bs9 and Bs5 chromosomes in relation to chromosome Bd4 could be the result of a Robertsonian rearrangement. Moreover, comparison with Bd4 revealed an altered order of the 5S rDNA locus and the clone Bd4L/6 on the short arm in relation to the centromere and Bd4L/7 on the long arm in Bs5. This suggests the occurrence of a probable pericentric inversion that determines the orientation of these regions in the Bd and Bs genomes (Figs 6A and 7A versus 6B and 7B). Similarly to the pattern of the Bd3-derived clones, all BACs in *B. hybridum* (Figs 6C and 7C) that had originated from chromosome Bd4 showed a distribution that was identical to those observed in its putative progenitors *B. distachyon* and *B. stacei*.

## DISCUSSION

### Reliable chromosome identification in *Brachypodium* annuals

Determining chromosome number, size and shape is usually a starting point for more advanced studies on the organization of a karyotype, but usually there are limitations in terms of reliable chromosome identification. This is particularly so for species that have numerous, structurally uniform chromosomes with none or few chromosome-specific markers (Dydak *et al.*, 2009; Kolano *et al.*, 2016; Susek *et al.*, 2016). *Brachypodium stacei* and *B. hybridum* have higher numbers of chromosomes compared with *B. distachyon*, most of which being small and similar in morphology and therefore difficult to identify. The morphometric parameters of the *B. distachyon* karyotype (Supplementary Data Table S1) are distinct enough to provide a reasonably reproducible identification of most of its chromosomes using simple DAPI staining (Fig. 1A), which can be enforced using a 25S rDNA probe to ensure a more reliable discrimination between the Bd5 and Bd4 chromosomes. However, even the use of a centromeric probe does not permit a good discrimination of Bd2 from Bd3 since these two metacentrics are almost identical in length and shape (Fig. 1B). With the exception of one pair of 35 rDNA-bearing chromosomes, no homologous pairs could be identified in *B. stacei* based on morphometric criteria. Specific morphometric characteristics of the Bs-genome chromosomes (Supplementary Data Table S2), especially in highly condensed spreads, may even hamper the localization of the centromeres, which are useful for identifying some chromosomes after DAPI staining (Fig. 1C); this can be resolved by using a centromeric probe (Fig. 1D). As was expected in *B. hybridum*, only a few pairs of larger chromosomes that had originated from genome Bd of a *B. distachyon* ancestor could be reliably distinguished on morphometric examination (Supplementary Data Table S3). Though exceptionally good chromosome spreads may produce satisfactory centromere visibility (Fig. 1E), FISH with a centromere-specific probe is usually indispensable in determining the exact chromosome shape of the small Bs-genome chromosomes in the complement (Fig. 1F). A clear difference in chromatin condensation within the two pairs of 35S rDNA sites, one of which is characterized by the presence of distended secondary constrictions and the other by high compaction (Fig. 1F), is connected with the occurrence of nucleolar dominance in this genotype of *B. hybridum*. This enigmatic phenomenon results in the expression of only the 18S-5.8S-25S rRNA genes inherited from the *B. distachyon* progenitor (Idziak and Hasterok, 2008; Borowska-Zuchowska *et al.*, 2016; Borowska-Zuchowska and Hasterok, 2017).

In large plant genomes that are saturated with ubiquitous repeats, the BAC–FISH-based approach either fails or is technically challenging and only partially successful in providing landmarks that are reproducibly diagnostic for individual chromosomes (Ma *et al.*, 2010; Majka *et al.*, 2017). However, as has already been demonstrated for several taxa, it is considerably more effective in plants with small and preferably sequenced genomes and also often in their close relatives, e.g. *Phaseolus vulgaris* (Fonseca *et al.*, 2010), *Brassica* (Xiong and Pires, 2011), *Solanum* (Szinay *et al.*, 2012) and recently even in the extremely small chromosomes of some aquatic (Hoang and Schubert, 2017) and carnivorous (Tran *et al.*, 2017) plants. Although there have been substantial and diverse cytomolecular studies performed in *Brachypodium* in recent years (for review see Hasterok *et al.*, 2015; IBI, 2014), no systematic research on chromosome identification had been reported in this genus. Since basic morphometric analyses have been ineffective in identifying almost all of the chromosomes in *B. stacei* and most of them in *B. hybridum*, we performed mcFISH-based karyotyping with consecutively applied sets of chromosome-specific markers, most of which were *B. distachyon*-derived BACs. Here, this approach provided reliable and reproducible identification of all of the chromosomes in *B. distachyon* (Figs 2A, D and 3A) and nine out of ten chromosome pairs in *B. stacei* (Figs 2B, E and 3B). Although the set of mcFISH landmarks that we used does not permit the effective discrimination of chromosome Bs2 from Bs9, in most of chromosome spreads they are sufficiently different in size and shape to be identified with the support of a morphometric evaluation both in *B. stacei* and in *B. hybridum* (Supplementary Data Tables S2–S3). The fact that both the number and chromosomal distribution of all of the markers in the karyotype of *B. hybridum* (Figs 2C, F and 3C) is a simple composite of the hybridization patterns that have been observed in its putative ancestors suggests that no major chromosome rearrangements have occurred within the genomes of these three species after the divergence of *B. hybridum*. Such long-lasting genome stasis is not always the case in other allopolyploids (e.g. Adams and Wendel; 2005, Renny-Byfield and Wendel, 2014).

### Grass and *Brachypodium* karyotype evolution

The Poaceae comprises >10 000 species, many of which are of key ecological and economic importance. Grass genomes show a great variety in their nuclear genome organization and plasticity, which at the cytomolecular level are connected with differences in the number, size, shape and sequence composition of the chromosomes that constitute their karyotypes. These features may vary even among closely related species and may provide useful information about various evolutionary rearrangements (Schubert and Lysak, 2011). It is now widely accepted that the most important mechanisms that shape the structure of plant genomes at the chromosomal level involve both the changes that affect entire sets of chromosomes (whole-genome duplications [WGDs] and allopolyploidization followed by subsequent diploidization) and individual chromosomes (aneuploidization and dysploidy), which are complemented by some minor inter- and intrachromosomal rearrangements (Schubert, 2007; Soltis *et al.*, 2015; Alix *et al.*, 2017; Mandakova *et al.*, 2017). Furthermore, recent comparative phylogenomic and cytomolecular studies of a larger number of species have suggested various modes of DNA double-strand break repairs to be another cause of the variability and evolution of genome size and karyotype (Schubert and Vu, 2016).

The technological advances of high-throughput DNA sequencing have greatly promoted both large- and small-scale comparative genomics, thereby providing new opportunities to reconstruct *in silico* the crucial events of angiosperm genome evolution that occurred in their extinct ancestors (Murat *et al.*, 2010; Salse, 2016*a*). A recent palaeogenomic study indicated that the ancestral grass karyotype (AGK) was structured into seven protochromosomes that contained 8581 protogenes with a minimal gene-space physical size of 33 Mb (Murat *et al.*, 2017). This extinct ancestor underwent a palaeotetraploidization event followed by diploidization via a number of reciprocal translocations, including ‘insertional’ centromeric fusions (nested chromosome fusions; NCFs) and end-to-end telomeric chromosome fusions (TCFs), which resulted in a 12-chromosome intermediate ancestral grass karyotype (Salse, 2016*b*). Nested chromosome fusions are common in grasses and have had a significant impact on the divergence of their karyotypes, which only seem to occur occasionally in eudicots (Fonseca *et al.*, 2016), in which TCFs are considered to be the key drivers of chromosome number reduction, such as in *Arabidopsis* and other crucifers (Lysak *et al.*, 2006; Mandakova *et al.*, 2017). Interestingly, among the modern grass karyotypes that have been studied, this of rice remains the most evolutionarily unchanged and thus resembles the putative post-tetraploidization AGK. Conversely, other grasses have been subject to various numbers of reshuffling events, which have resulted in more complex karyotypes (Murat *et al.*, 2017).

Nested chromosome fusions have also played a prominent role in the divergence of the *B. distachyon* genome. Comparative sequence analyses with rice and sorghum indicate that the present-day *B. distachyon* karyotype derives from a 12-chromosome AGK via a descending dysploidy that involved seven major NCFs, which resulted in *x* = 5 chromosomes (Abrouk *et al.*, 2010; IBI, 2010). Considering the 2-fold higher number of considerably smaller chromosomes that are present in the *B. stacei* genome (*x* = 10), it was anticipated that a lower number of NCFs would occur in this more evolutionarily ancient genome, which we aimed to verify by the direct comparative visualization of putative karyotype rearrangements in the Bd and Bs genomes.

Considering the findings of the sequenced-based modelling of the evolution of the grass karyotype, chromosome Bd3 of *B. distachyon* resulted from two separate NCFs that occurred via the reciprocal translocation of three ancestral chromosomes, which are equivalent to the rice chromosomes Os2, Os8 and Os10 (IBI, 2010). In Bd3, the most distally located Os2 segment is interrupted by the nested insertion of Os8 and the most internal by Os10 (Fig. 5A). In both *B. stacei* (Fig. 5B) and *B. hybridum* (Fig. 5C), chromosome Bs4 is hallmarked by clones Bd3S/1–3 on its short arm and Bd3L/10–14 on its long arm and corresponds to Os2 in its entire length, which, when combined with the results of palaeogenomic analyses, may indicate its structural conservation. Conversely, the arrangement of BACs along Bs6 reveals that its distal parts, which harbour the clones Bd3S/4–6 and Bd3L/9, respectively, correspond to two discontinued Os8 segments. Then, the internal part of this chromosome, which is delimited by clone Bd3S/7 on the short arm and Bd3L/8 on the long arm, corresponds to the Os10 equivalent of the rice chromosome. Thus, the structure of Bs6 is a relic of an ancient Os8/Os10 NCF that contributed to the early reduction of the original chromosome number from *x* = 12, as was suggested for the AGK-carrying intermediate ancestor (IBI, 2010). Importantly, Os8/Os10 and Os2 ancestral segments, which are consistently mapped on two different chromosomes of a given genome, were also found in several diploid and allopolyploid *Brachypodium* perennials, such as *B. pinnatum* (2*n* = 18 and 28), *B. sylvaticum* (2*n* = 18) and *B. phoenicoides* (2*n* = 28) (Idziak *et al.*, 2014; J. Lusinska, unpubl. res.). It seems likely, then, that the second NCF, which combined the fused Os8/Os10 segments (contemporary Bs6 in *B. stacei* and *B. hybridum*, perennials) with Os2 (contemporary Bs4 in *B. stacei* and *B. hybridum*, perennials) to assemble the present-day Bd3, occurred after the *B. distachyon*–*B. stacei* split, which recent comparative plastome analyses estimate to have happened 10.1 Ma (Sancho *et al.*, 2017).

It is assumed that chromosome Bd4 of *B. distachyon* results from a descending dysploidy that involved the NCFs of the terminally located Os12, Os9 and Os11 ancestral equivalents of the rice chromosome, which in Bd4 has the most internal position (Fig. 7A) (IBI, 2010). Contrary to inference from the palaeogenomic analyses, both Bs9 and Bs5 in *B. stacei* (Fig. 7B) and in *B. hybridum* (Fig. 7C) consist of all three linearly arranged Os11, Os9 and Os12 segments. The specific distribution of BAC markers along Bs9 and Bs5 supports the presence of a Robertsonian rearrangement, the possibility of which was already inferred from our previous observations on both mitotic (Hasterok *et al.*, 2006*b*) and meiotic (Betekhtin *et al.*, 2014) chromosomes. However, this was difficult to prove due to the sparsity of the physical map. Preliminary results on the Bd4-derived clone distribution on the chromosomes of the diploids *B. sylvaticum* and *B. pinnatum* and of the allopolyploids *B. pinnatum* and *B. phoenicoides* show that this rearrangement is only present in the Bs genome. In all species without this genome, their corresponding chromosomes reveal a very conserved composition, which seems to be identical to that of Bd4 (J. Lusinska, unpubl. res.). This implies that the NCFs that had assembled the Bd4-like chromosomes may be evolutionarily more ancient than the Bs-specific Robertsonian rearrangement, and it is probably a centric fission that split chromosome Bd4 into the Bs9 and Bs5 chromosomes. Moreover, since this rearrangement also involves Bs9 and Bs5 in *B. hybridum*, it must have occurred before the formation of this allotetraploid ~1 Ma (Catalán *et al.*, 2012).

### Conclusions

Based on the results of comparative mapping of BACs from two of the five *B. distachyon* chromosomes, although it would be premature to extrapolate to the phylogenies of the *Brachypodium* genus, our results show that genomes Bd and Bs appear to be cytogenetically the most divergent from each other within the genus. Mapping the Bd3- and Bd4-derived BACs in the Bs genome revealed various and complex rearrangements that influence the karyotypes in terms of both the number and structure of the chromosomes. Moreover, although *B. hybridum* is not a recent allotetraploid, it seems that no major chromosome rearrangements occurred in the Bd and Bs genomes of both the putative ancestors and their allotetraploid since the time of its formation. Mapping of both *Brachypodium* annuals and perennials based on CCB, using clones that are derived from all of the Bd-genome chromosomes, may provide a more comprehensive picture of the structure and evolution of the karyotype. Eventually, when combined with the results of further molecular phylogenetic studies and, in particular, of the ongoing whole-genome sequencing projects, this may contribute to resolving the enigmatic phylogenetic relations within the genus.

## SUPPLEMENTARY DATA

Supplementary data are available online at https://academic.oup.com/aob and consist of the following. Tables S1–S3: sets of morphometric data for the *B*. *distachyon*, *B*. *stacei* and *B*. *hybridum* chromosomes, respectively, which were analysed in this study using mcFISH karyotyping (shown in Fig 2). Figure S1: comparative mapping of the Bd4-derived clones in the different genotypes of *B. stacei* and *B. hybridum*.

Supplementary Table S1

Supplementary Table S2

Supplementary Table S3

Supplementary Figure S1
