# Supplementary figures and images for: Chromosome identification and reconstruction of evolutionary rearrangements in Brachypodium distachyon, B. stacei and B. hybridum
Source: Ann Bot. 2018 Jun 8;122(3):445–59. doi: 10.1093/aob/mcy086 (PMC6110338; doi:10.1093/aob/mcy086)

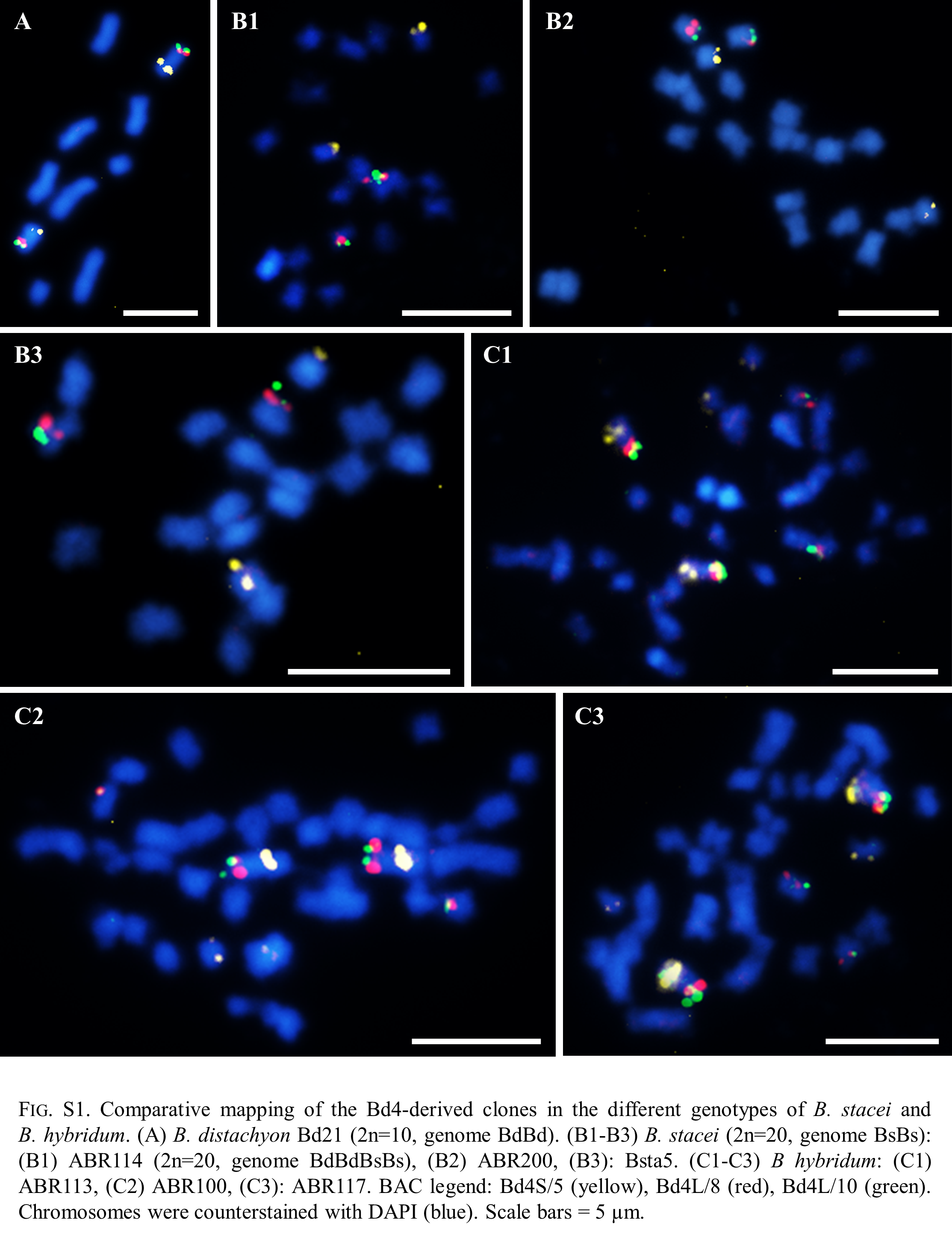

Supplement: Supplementary Figure S1 [file mcy086_suppl_aob-17959-s01.png]
